# Recent Advances in Transmission Electron Microscopy for Materials Science at the EMAT Lab of the University of Antwerp

**DOI:** 10.3390/ma11081304

**Published:** 2018-07-28

**Authors:** Giulio Guzzinati, Thomas Altantzis, Maria Batuk, Annick De Backer, Gunnar Lumbeeck, Vahid Samaee, Dmitry Batuk, Hosni Idrissi, Joke Hadermann, Sandra Van Aert, Dominique Schryvers, Johan Verbeeck, Sara Bals

**Affiliations:** 1 EMAT, University of Antwerp, Groenenborgerlaan 171, Antwerp 2020, Belgium; giulio.guzzinati@uantwerpen.be (G.G.); thomas.altantzis@uantwerpen.be (T.A.); maria.batuk@uantwerpen.be (M.B.); annick.debacker@uantwerpen.be (A.D.B.); gunnar.lumbeeck@uantwerpen.be (G.L.); vahid.samaeeaghmiyoni@uantwerpen.be (V.S.); dmitry.batuk@gmail.com (D.B.); hosni.idrissi@uantwerpen.be (H.I.); joke.hadermann@uantwerpen.be (J.H.); sandra.vanaert@uantwerpen.be (S.V.A.); nick.schryvers@uantwerpen.be (D.S.); jo.verbeeck@uantwerpen.be (J.V.); 2 Institute of Mechanics, Materials and Civil Engineering, Université catholique de Louvain, Louvain-la-Neuve 1348, Belgium

**Keywords:** TEM, electron diffraction tomography, STEM, atom counting, electron tomography, compressed sensing, EDS, EELS, nanomechanical testing, ACOM TEM

## Abstract

The rapid progress in materials science that enables the design of materials down to the nanoscale also demands characterization techniques able to analyze the materials down to the same scale, such as transmission electron microscopy. As Belgium’s foremost electron microscopy group, among the largest in the world, EMAT is continuously contributing to the development of TEM techniques, such as high-resolution imaging, diffraction, electron tomography, and spectroscopies, with an emphasis on quantification and reproducibility, as well as employing TEM methodology at the highest level to solve real-world materials science problems. The lab’s recent contributions are presented here together with specific case studies in order to highlight the usefulness of TEM to the advancement of materials science.

## 1. Introduction

Transmission electron microscopy (TEM) is a very rapidly developing field. The scope, breadth of information, and power of the various types of TEM techniques are being expanded every year. A modern TEM constitutes a complex characterization facility, capable of collecting diffraction patterns from volumes of a few cubic nanometres in size and imaging samples down to the atomic scale. When combined with tomography, a technique which derives three-dimensional (3D) information from two-dimensional (2D) images, one is able to determine the structure and shape of nanostructures in 3D, even with atomic resolution [1,2]. More than this, TEM allows performing spectroscopies that can analyse, once more down to the atomic scale, the composition of the sample, but also bonding, optical, and electronic structure properties both in 2D and 3D. Finally, a growing range of experiments can now be performed in situ in the TEM with simultaneous characterization, offering valuable insight in a variety of processes.

For this special issue on Materials Science in Belgium, we will focus on the contribution of the EMAT research group located at the University of Antwerp. EMAT is the foremost electron microscopy laboratory in Belgium, and one of the leading electron microscopy groups in the world, currently composed of more than 50 researchers and equipped with a wide range of state of the art instrumentation. Already the name, a contraction of electron microscopy for materials science, reveals the strong focus on materials, reflected also in the dense network of national and international collaboration with leading groups. Every characterization technique that is available here can be applied at the state of the art, and EMAT constantly strives to develop them further and push the boundaries of what is possible.

In this paper, we showcase a selection of examples of applied TEM as well as of instrumental developments that were contributed by EMAT to the field of materials science. We show how TEM can recover the different types of information from different materials, the methodological and instrumental advances pioneered by this lab, and case studies that showcase the determinant role of electron microscopy in solving different materials science problems.

## 2. TEM as a Structural Characterization Tool

In functional materials, a deep understanding of the relation between the properties and the crystal structure is the key to designing new materials and improving existing ones. TEM provides unique opportunities for crystal structure analysis at a very local scale, which, in many ways, complements powder diffraction techniques, such as X-ray or neutron powder diffraction, where the data is collected from a comparatively large volume of the material. TEM can access structural data both in reciprocal and direct space. Electron diffraction (ED) patterns contain information on the symmetry of the crystal and, in some cases, the intensities of the reflections can be used quantitatively to solve the crystal structure [3]. Coupling of scanning transmission electron microscopy (STEM) with atomic resolution spectroscopic techniques, such as energy-dispersive X-ray spectroscopy (EDS) and electron energy loss spectroscopy (EELS), allows analysis of the chemical composition, oxidation state, and coordination number of the individual atomic columns in the structure. In this section, we will give some examples of how these techniques were used to unveil structures that were out of reach for powder diffraction techniques due to different reasons.

Some types of samples can be difficult to study with powder diffraction, but are excellent for study using TEM, for example multiphase samples, samples with local defects and modulated materials [4,5,6,7]. While often such materials can still be analysed with powder diffraction, the data might be difficult for interpretation due to a variety of factors such as the presence of a large number of reflections, reflection overlap and anisotropic reflection broadening. There are clear advantages to the use of TEM: as electrons in a TEM interact much more strongly with matter than X-rays or neutrons, volumes of a few tens of nm^3^ are already enough to provide a clear diffraction pattern which is then from a single crystal, while images of the structure in direct space can already be taken with only a few atoms. As an extra advantage, the electron wavelength is a few orders of magnitude smaller than that of typical X-rays or neutrons, increasing the radius of the Ewald sphere (which is equal to the wave vector) and making its curvature almost flat, thus making ED patterns (almost) two-dimensional sections of the reciprocal lattice of the crystal [8]. As a result, ED provides easily interpretable information about the symmetry of the crystal, on a scale ranging from micrometres down to nanometres. The two-dimensional sections can also be combined to reconstruct the three-dimensional reciprocal lattice, using, in principle, simple pen-and-paper [3].

Structure solution and refinement from TEM has proven especially useful for such prominent materials as cathode materials for lithium-ion batteries. Understanding structure transformations upon reversible intercalation (during discharge) and de-intercalation (during charge) of the Li^+^ or Na^+^ ions is crucial for the improvement of the capacity, charge density, and lifetime of the battery. By design, batteries are multi-component devices, where the cathode materials are primarily used in powder form to ensure a homogenous mixing with amorphous additives (carbon) as electric conductors and with an electrolyte. This limits the possibilities of powder diffraction for the analysis of the structures. As ED patterns can be collected from single-crystal particles mere nanometres in diameter, the powder mix is effectively a collection of single-crystal samples for ED. On lithium battery materials, EMAT’s most important contributions through TEM are based on the use of electron diffraction tomography (EDT) or STEM images.

The specific advantage of EDT for lithium battery materials stems from the higher sensitivity of electron diffraction to elements with small atomic number (Z), such as lithium, compared to X-ray diffraction, as well as the single-crystal nature of the electron diffraction data [9,10]. However, direct structure solutions from ED patterns are hampered by multiple scattering of the electrons on their path through the crystal, even in samples with a thickness of only a few nanometres [11]. This makes the intensities of the reflections very dependent on the thickness and orientation of the crystals, resulting in intensities that deviate significantly from the intensities expected in the kinematic (i.e., single scattering) approximation. The detrimental effect of multiple electron scattering can be mitigated using either precession electron diffraction (PED) [12] or electron diffraction tomography. Both techniques result in off-zone patterns (although in the case of precession electron diffraction many off-zone patterns are summed and recombined into a seemingly in-zone pattern) which decreases the amount of possible multiple scattering paths and, thus, results in intensities closer to the kinematic ones (and, thus, called quasi-kinematical) [13]. Both techniques have been usefully applied for determining the structures of lithium battery cathode materials. In PED (whose applications are also explored in Section 8.1 of this paper) the crystal is tilted in the zone axis orientation, then the incident electron beam is tilted slightly (~1°) off the optical axis and rocked azimuthally around the optical axis, while keeping the tilt by using electromagnetic coils. This change in the direction of incidence produces the off-axis patterns. A second set of coils placed after the sample cancels out the beam tilt recombining the different patterns together. PED was used to solve and refine, for the first time, the structure of Li_2_CoPO_4_F [14]. Currently, the EDT technique (combinable with PED) is more often used because of its finer sampling of the reciprocal space [15], as simple PED only provides a few diffraction patterns and, hence, only a few slices of reciprocal space, while EDT probes a large fraction of the reciprocal space. In EDT, the sample holder is tilted inside the TEM column and ED patterns are collected using an increment of ~1°, avoiding major crystallographic zone axes. The acquired ED patterns are used as an input for a reconstruction algorithm, which produces a 3D dataset containing quasi-kinematical diffraction intensities. They can be used for crystal structure solutions by the conventional methods of single-crystal X-ray crystallography. Using this technique, many structures of cycled cathode materials were already successfully solved and refined. For example, for cycled AVPO_4_F (A = K, Li) the results of EDT revealed that K is not completely extracted from the charged material and that Li in the discharged material occupies new crystallographic positions. Hence, the K atoms in this material not only act as mobile species, but also as structural pillars supporting the VPO_4_F framework in the charged material [16]. In the Li_2_FePO_4_F compound, prepared by electrochemical substitution of Na in LiNaFePO_4_F by Li, the EDT structure analysis unveiled the origin of capacity fading in the material. Removal of Li upon charging creates “dangling” P−O bonds resulting in a substantial bond imbalance, which is compensated through Fe migration towards the Li positions giving rise to Li/Fe anti-site disorder [17]. In LiRhO_2_, the EDT study showed that the layered structure transforms upon charging into a tunnel structure with rutile and ramsdellite channels [18].

Another crucial technique for lithium battery cathode materials is STEM [19,20]. TEM and STEM can both be used to visualize the atomic arrangement of the structure. The development of aberration-corrected TEMs has enabled imaging the atom columns with a resolution of 50 pm [21]. The first TEM method capable of atomic resolution was high resolution (HR) TEM imaging, where elastic interaction of coherent parallel electron beam with the crystalline lattice of the material results in an interference pattern (HRTEM image) that has the same periodicity as the crystal and can be used to retrieve information on the symmetry [22]. Inelastic interaction provides, due to the chromatic aberrations in the lenses, just a featureless background that reduces the images’ signal to noise ratio. The direct interpretation of these images is hindered by high sensitivity of the interference pattern to the sample thickness and the exact defocus of the microscope. Nevertheless, this method has been widely used for crystal structure visualization [23,24,25]. During the last two decades, atomic resolution STEM is more often used for this purpose, mainly due to the fact that it is more robust to the variations in the experimental settings and, thus, HR-STEM images are easier to interpret than HR-TEM images. In this method, a focused electron beam is scanned over the specimen and the transmitted electrons are collected by (typically) annular detectors below the sample. Depending on the acquisition settings of the microscope, there are two main STEM imaging modes useful in structure analysis. In the so-called high angle annular dark field STEM (HAADF-STEM) mode, the intensity of the acquired projection images is proportional to the average atomic number of the projected column (I~Z^2^) and scales with the thickness of the specimen, hence delivering chemically-sensitive information [26]. While in some case even light elements can be detected by HAADF-STEM [27], many materials combine elements with widely different atomic numbers. As a result, the contrast in the images is excellent for the high Z elements, but very poor for the low-Z ones and, in most situations, the accurate localization of the light elements is impossible due to the finite signal to noise ratio. The information from atomic columns of light elements, such as O, or even H, can be more easily obtained using another STEM-imaging mode, called annular bright-field STEM (ABF-STEM) [28,29,30,31].

**Figure 1:**
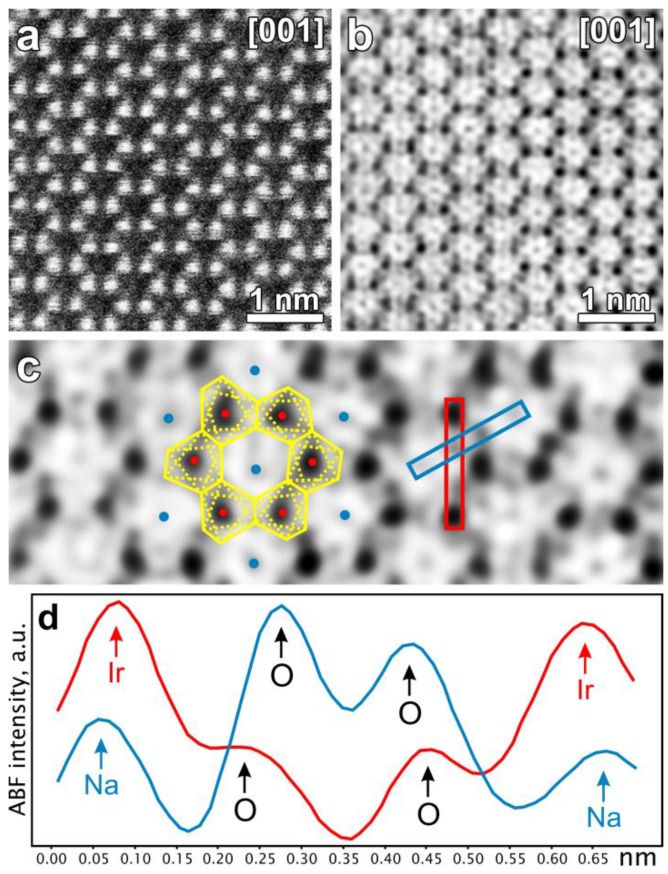
Complementary [001] HAADF-STEM (**a**) and ABF-STEM (**b**) images of fully-charged Na2-xIrO3 with an O1-type structure. Magnified fragment of the ABF-STEM image (**c**) with marked projections of the IrO6 octahedra (red dots: Ir columns; blue: Na; and yellow lines: O octahedral projections). Intensity profiles measured in the areas marked in blue and red highlighting short and long projected O−O distances (**d**). Adapted with permission from [33]. Copyright 2016 American Chemical Society.

Atomic-resolution HAADF-STEM and ABF-STEM imaging, for example, revealed the crystal structure transformation of the model battery cathode materials Li_2_IrO_3_ and Na_2_IrO_3_ [32,33]. These compounds belong to a family of Li, Na-rich layered rock-salt compounds, which demonstrate capacities larger than those expected for the pure cationic redox activity. STEM imaging showed the formation of short O-O distances, confirming that the excess capacity is due to participation of both cationic and anionic sublattices (Figure 1). HAADF-STEM imaging can be used to analyse the migration of the transition metal cations into the Li positions upon cycling, as it was done for Li-rich Li_2_Ru_1−y_Ti_y_O_3_ phases. This migration is only partially reversible during the discharge, leaving a fraction of the transition metal cations trapped in the tetrahedral sites, hence resulting in a gradual voltage fade of the material [34]. In LiRhO_2_, the HAADF-STEM images could be used to infer a transition mechanism from layered into a 3D structure, which involves local migration of Rh and O species [18]. Sometimes, the defect analysis using real space imaging can greatly facilitate interpretation of the powder diffraction data. In the Li_3_Ru_y_Nb_1−y_O_4_ family of rock salt structures, which was used to test the limits of the anionic redox activity [35,36], the (Nb,Ru)O_6_ octahedra form either zigzag, helical, jagged quasi-1D chains, or 0D clusters depending on the Nb/Ru concentration. The HAADF-STEM images unveiled a plethora of extended planar defects, which give rise to anisotropic broadening of reflections in the powder X-ray diffraction. Modelling these defects significantly improved the crystal structure refinement.

Another type of materials where TEM has proven to be indispensable for structure solution is modulated materials, whether commensurately or incommensurately modulated. Often the satellites or superstructure reflections are weaker compared to the parent cell reflections and form a very dense set of reflection in the powder diffraction patterns, which complicate the Rietveld refinement. Using ED, one can take clear two dimensional sections through the reciprocal lattice to determine the cell parameters and symmetry, with clearly separated satellite reflections in most cases. This can be complemented with a direct view of the structure in direct space using high-resolution TEM or STEM techniques. Structure models for numerous modulated materials were solved using this combination of techniques, most frequently followed by subsequent refinement using powder diffraction data. Examples range from scheelites [37] to perovskite-based structures [38]; a description of the typical solution route has already been published in a previous review paper by Batuk et al. [4].

When the materials are sufficiently stable under the electron beam (the doses for a high resolution spectroscopic map are in the order of few pC/Å^2^), the diffraction patterns and images can be complemented with high-resolution EDS or EELS maps, allowing to support the structure models with direct knowledge on the distribution of the elements over the different solved atomic positions [39]. This was necessary, for example, to determine the structure at the interface between a perovskite substrate and fluorite film, which showed unexpected features in the STEM images. The STEM images showed where the atoms were, but only the HR-EDS and EELS studies demonstrated which elements were at those positions, revealing a redistribution of the cations over several layers at the interface [40]. The vast majority of the crystalline solid-state materials are indeed stable under the electron beam and the use of high-resolution TEM imaging provides a still image of the atomic arrangement. Some materials are too beam-sensitive, and even a short exposure to the electron beam (doses for a high-resolution image are of the order of fC/Å^2^) decomposes them or renders them amorphous, thus requiring special procedures for their TEM analysis [41]. However, there is a narrow group of materials, in which the energy transfer from the electron beam triggers interesting structure transformations. This approach has been used to analyse the chemistry and dynamics of lone pair Bi^3+^ cations in the layered Bi_3n+1_Ti_7_Fe_3n−3_O_9n+11_ perovskite-anatase intergrowth materials with variable thickness of the perovskite layers [42,43] (Video S1). The lone pair cations are prone to an asymmetric coordination environment, which can induce electric polarization of the material. The HAADF-STEM and ABF-STEM data revealed that in the Bi_3n+1_Ti_7_Fe_3n−3_O_9n+11_ family, the Bi^3+^ cations trigger off-centre displacements of the transition metal cations. Although the materials are antiferroelectric, the exact pattern of polar atomic displacements depends on the thickness of the perovskite blocks. The dynamic changes in the local configuration of the structure, e.g., upon absorbing energy from the electron beam, occur cooperatively, so that a large number of atoms change their position at the same time, which could be recorded in real-time using a fast HAADF-STEM acquisition.

## 3. Quantitative Imaging in Transmission Electron Microscopy

**Figure 2:**
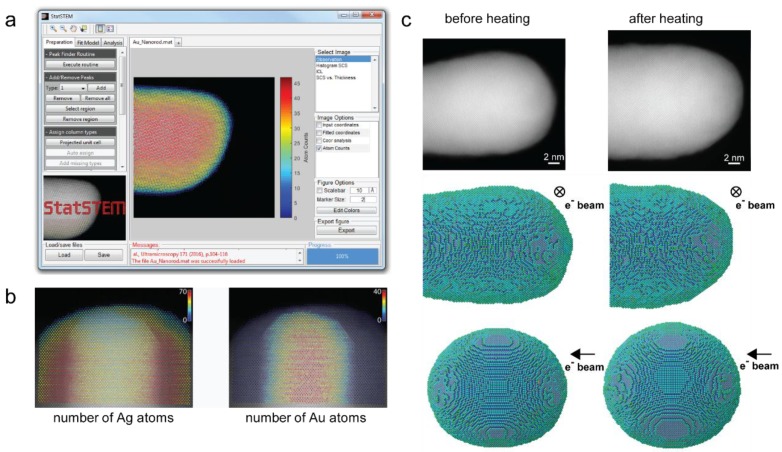
(**a**) Screen shot of the StatSTEM software, showing the atom-counting results on an experimental image of a Au nanorod; (**b**) Counting the number of atoms from a HAADF-STEM image of a Ag-coated Au nanorod; and (**c**) 3D atomic scale characterization of a Au nanodumbbell before and after heating.

Although high-resolution HAADF-STEM imaging is considered nowadays as a standard technique for the structural and compositional characterization of different nanomaterials at the atomic scale, a quantitative analysis is often very challenging. Since the beginning of the decade, a great deal of effort has been put on the development of approaches to reliably quantify ((S)TEM) data [44]. The main objective is to extract precise and accurate numbers for unknown structure parameters including atomic positions, chemical concentrations, and atomic numbers. In order to extract these quantitative measurements from atomic resolution (S)TEM images, statistical analysis methods are needed. For this purpose, statistical parameter estimation theory has been shown to provide reliable results [45]. In this framework, images are purely considered as data planes [46], from which structure parameters have to be determined using a parametric model describing the images. Atomically-resolved HAADF-STEM images are described with a parametric model in which the projection of an atomic column corresponds to a Gaussian peaked at the column’s position. The parameters of this model, including the atom positions, the height and the width of the Gaussian peaks, are determined using the least squares estimator [44,47,48]. As such, the positions of atom columns can be measured with a precision of the order of a few picometres [49,50,51,52,53,54,55], even though the resolution of the electron microscope is still one or two orders of magnitude larger. Moreover, small differences in average atomic number, which cannot be distinguished visually, can be quantified using HAADF-STEM images [44]. In addition, this theory allows one to measure compositional changes at interfaces [56,57,58], to count atoms with single atom sensitivity [59,60,61], and as we will see in a next part also to reconstruct atomic structures in three dimensions (3D) [1,59,62,63,64,65]. Making this well-established quantification method easier to apply for all scientists was the motivation behind the development of StatSTEM, a user-friendly software for the quantification of high-resolution STEM images (Figure 2a) [47]. Here, two recent applications of advanced quantification methods in atomic resolution STEM will be briefly discussed.

In HAADF-STEM, the image intensities scale with the thickness of the sample and with the atomic number (I~Z^2^). For homogeneous materials the scattering cross-sections, i.e., the quantified integrated intensities at the atomic column positions, only depend on the thickness and can, therefore, be used to count the number of atoms in each atomic column by either employing advanced statistical methods [60,61] or by comparison with simulations [66]. For heterogeneous materials, all types of elements will contribute differently to the scattering cross-sections, thus, significantly complicating atom-counting with respect to monotype nanostructures. In addition, small changes in the atom ordering in the column have an effect on the scattering cross-sections. In order to unscramble this information, an atomic lensing model was introduced which allows to predict scattering cross-sections as a function of composition, configuration, and thickness [67]. When using this model the number of both Ag and Au atoms could be counted from an experimental HAADF-STEM image of a Ag-coated Au nanorod, as demonstrated in Figure 2b. This type of atom-counting results can also be used to gain access to the 3D atomic structure, as will be extensively shown in part 5.

## 4. Investigation of Beam Sensitive Materials in a TEM

### Compressed Sensing

For all the wealth of information that TEM can provide about a sample, its usefulness in some fields is still limited by the beam-sensitivity of the samples, and a great deal of work in the field is devoted into obtaining the most information for the electron dose. Examples of this are the application of rigorous statistical methods to the quantification of noisy high-resolution images, and the use of compressed sensing (CS) approaches in the development of electron tomographic algorithms [68].

Compressed sensing is a signal processing approach which consists of expressing a signal into a mathematical basis where it appears sparse and, hence, can be described with less information. As such, this approach allows reconstructing a dataset from a limited amount of samples. Since experimental images have a high degree of natural sparsity, it is possible to apply these methods to STEM imaging with the aim of reconstructing an image by measuring only a fraction of the pixels, randomly distributed within the image frame.

**Figure 3:**
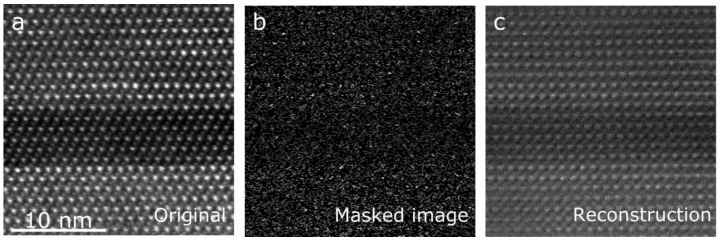
Experimental realization of compressed sensing for STEM imaging. (**a**) Conventional high resolution STEM image; (**b**) image over the same region of the same sample, where 20% of the pixels are acquired and 80% are blocked; and (**c**) the reconstructed image.

By using a specially developed fast electromagnetic beam shutter capable of operating at microsecond speeds, and synchronizing it with the microscope’s scan engine, it becomes possible to selectively illuminate only a selected number of pixels in the image [69]. Early experimental tests, where this technique was applied to the reconstruction of high-resolution images (Figure 3) or to the imaging of highly beam-sensitive samples [70], have been successful. While theoretical doubts have been raised on whether CS can beat conventional denoising algorithms on a purely statistical basis [71], the experimental results suggest that this method of reducing beam damage is effective beyond expectations, and that beam-sensitive samples can undergo healing processes that substantially limit beam damage when exposure is not continuous [70].

## 5. Atomic Resolution Tomography

While TEM and STEM are ideal techniques to investigate materials at both the nanometre and the atomic scale and have therefore been widely used in the study of nanomaterials, the obtained images only correspond to 2D projections of 3D objects [72]. Therefore, electron tomography, a technique which derives 3D information from 2D projections, can be used in order to obtain the necessary 3D information [73]. Most tomography results have addressed nanometre scale characterization, but recent advances in material sciences raised questions that could only be answered by the atomic scale 3D structures and have fostered the extension of the technique to the atomic scale.

**Figure 4:**
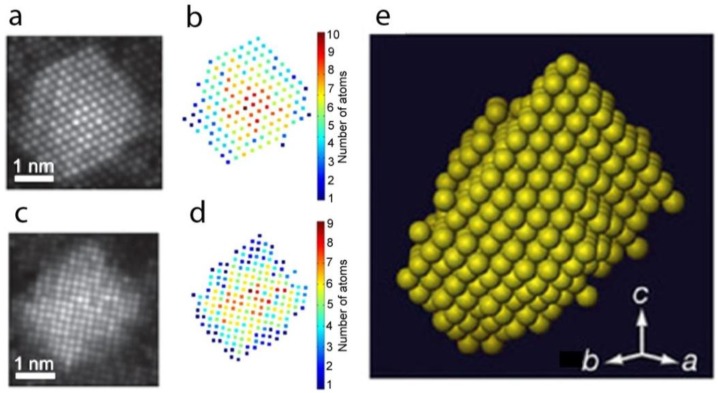
(**a**,**c**) Refined models for two HAADF-STEM projection images of a Ag atomic cluster embedded in an Al matrix; (**b**,**d**) Number of Ag atoms per projected atomic column for both images; (**e**) 3D representation of the reconstructed volume of the Ag nanoparticle, based on discrete tomography [59].

The first work in atomic resolution tomography, which is also considered as a breakthrough in the field, was published almost seven years ago by Van Aert et al. By combining atom-counting results (Figure 4b,d) obtained from images of a 3 nm Ag nanoparticle embedded in an Al matrix under different viewing directions (Figure 4a,c), the 3D space could be reconstructed for the first time at an atomic level using so-called discrete tomography (Figure 4e) [59]. Here, it was assumed that all atoms are positioned on a fixed face-cantered-cubic grid without any vacancies being present. The 3D reconstruction was compared to projection images that were acquired along additional zone axes and an excellent match was found, thereby validating the quality of the reconstruction. This technique, however, assumes that atoms are located on a fixed regular lattice, while deviations often occur because of defects, strain, or lattice relaxation and are of utmost importance as they determine the physical properties of nanomaterials.

**Figure 5:**
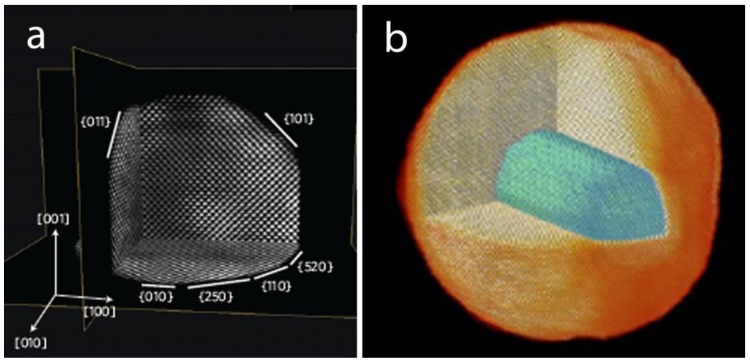
(**a**) Slices through the atomic resolution 3D reconstruction of a Au nanorod, revealing the atomic lattice and the surface facets present; (**b**) 3D visualization of a core—shell Au@Ag nanorod, where the Au core is rendered green, and the Ag atoms are visualized in orange [2,74].

One year later, a different approach was proposed by Goris et al. [2], which was applied to Au nanorods in order to visualize the atom positions in 3D. Hereby, four atomic resolution projection images were used as an input for an advanced reconstruction algorithm based on compressive sensing that exploits the natural sparsity deriving from the discrete atomic structure of the material, and does not require assumptions on the crystal structure [68]. Indeed, for high-resolution electron tomography, only a limited number of voxels are expected to contain an atomic core, and most voxels will correspond to vacuum. By exploiting this in the algorithm it is possible to create a reliable reconstruction of the atomic lattice from only a few projections [2]. The methodology enabled a precise determination of the surface facets of the investigated Au nanorod, as illustrated in Figure 5a.

In a subsequent work, the same approach was used by Goris et al. for the characterization of core–shell bimetallic Au@Ag nanorods, where five atomic resolution HAADF-STEM images were acquired (Figure 5b) [74]. In this case, since the intensities depend on the atomic number in the individual HAADF-STEM projections, a careful analysis of the intensities in the reconstructed volume allowed to distinguish between Ag and Au atoms. Therefore, the atomic structure at the core/shell interface could be reliably determined.

**Figure 6:**
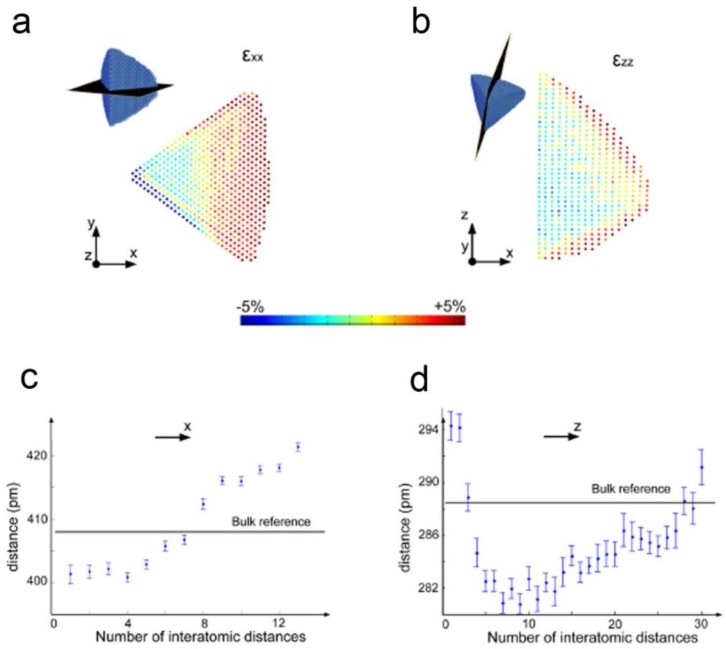
(**a**,**b**) εxx and εzz strain field showing surface relaxation in both directions. The colour scale indicates the expansion of the lattice parameter with respect to a reference; (**c**,**d**) The surface relaxation was confirmed by measuring the lattice parameter on slices through the reconstructions at the positions indicated in (**a**,**b**) [75].

While these approaches allowed to study the 3D atomic structure, they do not allow to determine the atomic coordinates with sufficient precision to three dimensionally study, e.g., strain. Recently, Goris et al., conducted a study to compare strain investigations relying on 2D projections with 3D measurements based on high-resolution electron tomographic reconstructions [75]. The 3D measurements displayed an outward relaxation of the crystal lattice which could not be seen from a single 2D projection image. This was done by acquiring a continuous tilt series of HAADF-STEM projections with a tilt increment of 2°, and applying a dedicated alignment procedure and reconstruction algorithm. Typically, during conventional alignment, the tilt angles are considered a fixed parameter, set to the nominal value used during acquisition. Here, the angles were optimized during the reconstruction in an iterative manner. As previously discussed, the outcome of a 3D reconstruction at the atomic scale is usually a continuous 3D volume of intensity values, from which the centre of each atom can only be determined by additional successive analysis. These datasets are very large, and in the absence of objective and automatic segmentation procedures it becomes difficult to obtain atom coordinates. Goris et al., could overcome this limitation by assuming that the 3D atomic potential can be modelled by 3D Gaussian functions. This assumption significantly simplifies the reconstruction problem, yielding the coordinates of the individual atoms as a direct outcome of the reconstruction. This highlights how using shape models can be a powerful tool to solve many problems in the field of electron tomography. The approach was applied to an Au nanodecahedron containing more than 90,000 atoms [75] and, since the atomic coordinates are a direct output of the reconstruction, it becomes straightforward to calculate the 3D displacement. The displacements were calculated with respect to a reference region in the middle of the segment. The εxx and εzz strain field components were obtained by computing the derivatives of the 3D displacement. Slices through the resulting εxx and εzz volumes extracted through the middle of the segment and the results are presented in Figure 6a,b.

From the previous section it is clear that tomography typically requires several images demanding a substantial electron dose. To circumvent this problem, atom-counting results from just a single projections can be used as an input to retrieve the 3D atomic structure [62,64,65,66]. In combination with prior knowledge about a material’s crystal structure, an initial 3D model is generated. Next, an energy minimization is performed to relax the nanoparticle’s 3D structure. This new approach opens up the possibility for the study of beam-sensitive materials, 2D self-assembled structures [64], and in conjunction with in situ experiments where the dedicated hardware makes tomography impossible. As an example this was recently applied by De Backer et al., as shown in Figure 2c, to retrieve the 3D atomic structure of a nanodumbbell on an in situ heating holder allowing only a limited tilt range [65]. From the reconstructions, the surface facets can be clearly observed for the entire tip of the nanostructure and a significant increase in low index facets is observed after heating which can be expected to be of critical importance for the study of catalysis.

## 6. Spectroscopic Techniques in a TEM

While as illustrated in the previous sections, (S)TEM can provide quantitative structural information in 3D for systems with one or two atomic species as long as the atomic number differs sufficiently, relevant samples for materials science contain often a much wider variety. The question is then how can we retrieve the distribution of different elements in the specimen and can we even extract bonding information. In order to answer these questions, spectroscopic techniques are an essential part of materials characterization methods.

EDS and EELS are spectroscopic techniques which are almost always present in modern TEMs. By using them in combination with STEM, a complete 2D mapping of the chemical elements in the specimen can be obtained. Recent advances in instrumentation, such as the implementation of electron monochromators, allow EELS edges to be acquired at energy resolutions close to that of synchrotron-based spectroscopies providing extra information which cannot be obtained by EDS. For example, investigating the core-loss region of a spectrum, can reveal information concerning the valency of cations, oxygen coordination, and bond elongation, with spatial resolutions going down to the atomic scale. Analysing electrons which have lost few eV, belonging to the so-called low-loss part of the spectrum, allows to study inter-band transitions and optical properties of nanomaterials.

### High-Resolution STEM-EELS

The combination of high-resolution imaging and high energy resolution spectroscopies has proven to be a formidable tool to solve materials science problems. While the former offers, with sub-angstrom resolution, structural information, including properties such as strain, the latter can provide high-resolution chemical composition, as well as oxidation state [76], and even bonding information, forming a compelling picture of the material’s properties, which can be particularly valuable in the case of localized systems such as interfaces.

**Figure 7:**
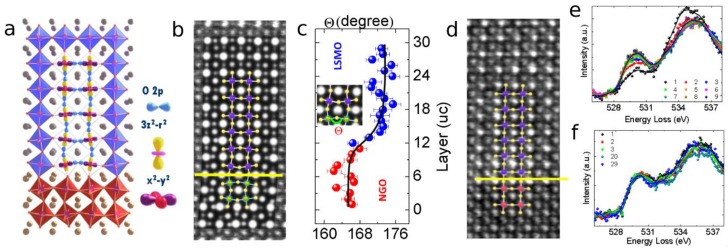
Octahedral tilt and orbital hybridization in a perovskite thin film. (**a**) At the interface between perovskites with different octahedral tilt systems, the oxygen octahedra have to rotate to maintain corner connectivity across the interface; (**b**) inverted-contrast ABF-STEM image showing the octahedral tilt in a LSMO on NGO film. In the inset, the image simulated from the obtained atomic structure (at the centre); (**c**) B-O-B angle between the oxygen atoms and the B cations as a function of the atomic plane; (**d**) inverted ABF-image of a similar film grown on top of a STO buffer layer, displaying no tilt; (**e**) the fine structure of the EELS oxygen peak measured in the buffered film. Each spectrum is measured from a region with a one unit cell thickness. The numbers in the inset indicate the distance of the chosen layer from the interface, expressed in unit cells. There is an obvious dependence of the fine structure from the distance; and (**f**) the fine structure of the EELS oxygen peak measured in the non-buffered film. There does not appear to be any dependence of the shape of the fine structure on the distance from the interface.

In particular, interfaces between oxides have been under intense study for the variety of novel emerging quantum phenomena they exhibit, depending on a variety of factors (strain, oxygen stoichiometry, cation intermixing, etc.). As an example, we can consider how the magnetic properties of epitaxial manganite films depend heavily on the chosen substrate. In the bulk case La_2_/3Sr_1_/3MnO_3_ (LSMO) is ferromagnetic with an easy magnetization axis along the b crystallographic direction and a Curie temperature of 350 K. However, when a thin film (of less than 20 unit cells) of LSMO is grown on an NdGaO_3_ (NGO) substrate, its Curie temperature drops dramatically (180 K for 10 unit cells). In such a very thin film the easy magnetization axis is oriented along the direction [77,78,79]. The main driving mechanism for this behaviour is oxygen octahedral coupling. Indeed, the oxygen octahedra in the film have to rotate in order to retain corner connectivity across the interface (Figure 7a), thus deviating from the natural tilt system of LSMO. In a state of the art microscope, these effects can be visualized and quantified by high-resolution STEM imaging (Figure 7b), determining the new tilt angle unit cell by unit cell and, thus, the extent of the perturbation (Figure 7c). This deviation has a dramatic effect on the properties of the material, as it affects the overlap between the atomic orbitals and therefore tuning the oxygen-mediated superexchange interaction between the B cations (Mn) which determine the magnetic properties of the material [79,80].

This can be effectively confirmed by observing the fine structure of the oxygen edge in high spatial and energy resolution EELS data (Figure 7f), which displays a pre-peak associated to the hybridization of the p-orbitals of the oxygen with the d-orbitals of the transition metal cations. The intensity of this prepeak proportional to the orbital overlap, is clearly reduced closer to the interface due to the octahedral tilt that reduces the overlap orbital, in agreement with the explanation. Understanding these effects allows to effectively tune and engineer them [61]. A thin (eight unit cells) buffer of SrTiO_3_ between the NGO substrate and the LSMO film effectively suppresses the octahedral tilt in the first LSMO unit cells, restoring the original structure of the LSMO layer (Figure 7d), whose properties are then much closer to the bulk material, as well as the fine structure of the oxygen peak (Figure 7e). Indeed, even a one unit cell buffer has a major impact [78,79].

## 7. Spectroscopic Tomography

As demonstrated earlier (Figure 5, atomic resolution tomography), the HAADF-STEM intensity, depending on both sample thickness and atomic number Z, allows studying of the chemical composition in 3D. However, it becomes very challenging to use HAADF-STEM tomography for samples where different elements are mixed, or the differences in atomic number Z are small. In such cases, tomographic methods need to be combined with spectroscopic investigations to determine in 3D not only the structure, but also chemical composition, and even oxidation.

### 7.1. EDS Tomography

EDS studies the characteristic X-rays which are emitted during the beam-sample interaction, and whose energies correspond to the difference between specific atomic energy levels. Since the number of generated X-rays scales with the thickness of the sample, a 2D elemental map can, in principle, serve as a projection image for electron tomography. With previous generation instruments, attempts to obtain 3D information by EDS were severely hindered by the directionality and inefficiency of the sample-detector geometry, as the EDS detector is typically placed at a specific angle from the specimen. Therefore, the optimal signal can only be collected when the sample is tilted towards the detector while at different tilt angles the sample holder can cause significant shadowing. New-generation detection systems where four X-ray detectors are placed symmetrically around the sample reduce the blocking of the generated X-rays [81]. Using this type of system allows to combine EDS with tomography, therefore, enabling the determination of the composition of complex nanostructures in 3D.

**Figure 8:**
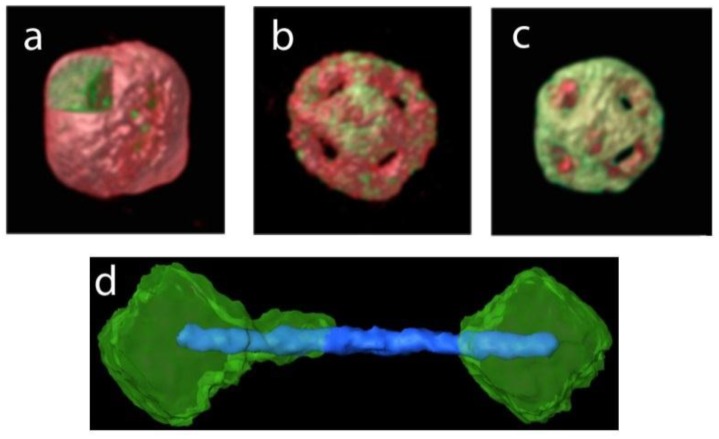
(**a**–**c**) 3D visualization of the EDS reconstructions of three different nanoparticles at different stages of a galvanic process reaction. Au and Ag are represented by red and green, respectively; (**d**) 3D visualization of the EDS reconstruction of a Fe-Co nanodumbbell, revealing the distribution of the elements in 3D. Co and Fe are represented by blue and green, respectively [82,83].

An early demonstration of the combination of EDS with tomography has been realized by Goris et al., in 2014. In the first work, compositional changes in nanoparticles containing Au and Ag could be determined, for different steps of a galvanic replacement reaction, Figure 8a–c [82]. Typically, during such a reaction, solid metal nanoparticles become hollow through oxidation of one metal by another with a larger reduction potential, so that the metal with the larger reduction potential gets deposited in the form of so-called nanocages. In the second work, the technique was used for the determination of the distribution of Fe (Z = 26) and Co (Z = 27) in Fe-Co hetero-nanostructures in 3D, Figure 8d [83].

Further steps are needed to move from analysing the elemental distribution, to actual quantitative data. A recent proposal is based on combining ratio maps, obtained from the so-called ζ-factor (zeta-factor) method [84], with thickness information extracted from a HAADF-STEM reconstruction of the same nano-object [85]. Conventional EDS quantification uses the Cliff-Lorimer method, originally developed for the investigation of thin foils [86]. However, tilting the sample varies the amount of X-rays which are absorbed by the sample itself, which is an important parameter for accurate EDS quantification, in turn hampering a straightforward quantitative 3D reconstruction. The ζ-factor method presents major advantages, since reliable ratio maps are obtained, not affected by shadowing effects.

### 7.2. EELS Tomography

Recently, in the work of Goris et al., it was shown that it is possible to visualize in 3D the distribution of Ce^3+^/Ce^4+^ in ceria nanoparticles, by combining monochromated EELS with electron tomography [87]. Initially, 2D maps of high energy resolution EELS data of the Ce edge were acquired from CeO_2−x_ nanoparticles along different tilt angles. Instead of analysing these maps and using the resulting valency maps as an input for a reconstruction algorithm, a different approach was followed, where complete EELS data cubes were used, leading to a 4D dataset from which an energy loss spectrum could be extracted for each reconstructed voxel. Although such experiments are challenging and computationally demanding, 3D quantitative results can be obtained in a more straightforward and reliable manner, since it prevents the accumulation of the errors introduced during the extraction of the 2D valency maps. The technique was applied at two different nanoparticles with different morphology, enabling the determination of the amount of Ce^3+^ at different crystallographic facets, which, in turn, provides a direct explanation of the observed lower catalytic activity between different surfaces.

## 8. TEM for Functional Characterization

While the TEM is the ideal tool to characterize the structure and chemical composition of a sample down to the atomic level, it can provide a wealth of information well beyond that, and characterize a variety of functional properties, such as optical, electronic, mechanical, magnetic, and more [88]. The techniques used to access these different properties can vary widely, from careful applications of more conventional electron spectroscopies, to in situ experiments realized through dedicated equipment. Here we will present recent advances at EMAT in nanomechanical testing, performed with a specialized holder with pN force sensitivity, as well as in the study of electronic and optical properties, performed with EELS.

### 8.1. Nanomechanical Testing in the TEM

Nanostructured materials—including thin films, nanocrystalline materials, nanocomposites and nanoporous foams and small-scale materials have shown remarkable variations in several mechanical properties, such as improved strength and toughness of nanocrystalline materials and the high strength of small-scale materials. These different properties have steered the transition from macro-scale engineering to micro/nano-scale technologies, e.g., now thin films are the basic components of micro/nanoelectronics and various industrial sensors are gradually being replaced by the cheaper micro-electro-mechanical systems (MEMS)-based devices and microsystems. However, such small components are often very delicate and, thus, their mechanical properties need to be properly investigated. Indeed, measuring the mechanical properties, ideally under in situ conditions, and understanding the governing deformation and failure mechanisms of such structures are the key issues to guide the design of more reliable nanocomponents.

**Figure 9:**
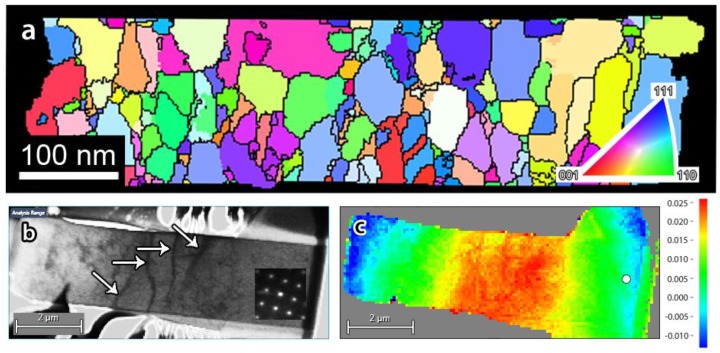
(**a**) Example of ACOM-TEM on a nanocrystalline Pd thin film. The colour code inverse pole figure is shown in the inset; (**b**,**c**) a virtual BF image and the corresponding local strain field map of a fatigued single-crystal Ni micropillar with dislocation walls seen as black curved lines running between opposite sides and are highlighted with white arrows.

While the excellent resolution of TEM allows to investigate defects in materials even down to the atomic scale, TEM investigations typically focus on very small volumes or individual defects, such as dislocations, interfaces, and so on [89,90,91,92,93], without being able to get good statistical data, essential to completely understand and/or model the behaviour of the nanomaterials. In order to bridge the gap between large area scanning electron microscopy (SEM) and atomic scale TEM, an automated crystal orientation mapping (ACOM) tool became recently available in EMAT [94]. This system is used to scan a region of a few µm^2^ and automatically indexes grains with a 1 nm resolution by template matching nanobeam diffraction patterns with calculated ones (Figure 9a). Furthermore, PED can be applied to obtain quasi-kinematical diffraction conditions to facilitate crystal orientation indexation yielding quantitative analysis on the granular level. Although similar to electron backscatter diffraction (EBSD) the spatial resolution is higher, the electron dose is significantly lower in comparison to the backscattering technique, making ACOM-TEM applicable to beam-sensitive materials as well. In the past, ACOM-TEM was applied mostly to investigate grain size distribution in nanocrystalline materials [95,96,97,98]. For example, Wang et al. [99] investigated grain boundary processes in nanocrystalline Pd after lab-on-chip tensile deformation. Nowadays, ACOM-TEM is used for in situ experiments, such as monitoring local phase changes during heating and/or grain growth during deformation using dedicated holders [100,101]. Furthermore, applying the accompanying TOPSPIN software, the recorded nano-diffraction patterns can be used to visualize the local strain field by measuring the change in the reciprocal lattice vectors [102]. For example, Figure 9b,c show the long range strain field due to the presence of dislocation walls in single-crystal Ni micropillar after fatigue tests.

New in situ TEM nanomechanical testing holders allow for a one-to-one relationship between loading-displacement data and direct TEM observation of the elementary plasticity and failure mechanisms. Conventional holders induce strain on a partially electro-polished metallic dog-bone sample with the size of a few millimetres [103]. An uncontrolled location of the hole as well as thickness variations in the sample make the loading conditions very complex. Therefore, such experiments are designed only for qualitative real-time investigation of microstructure evolution. For example, Tirry and Schryvers [104] used this holder to investigate stress-induced transformation of polycrystalline and single crystal sheets of Ni-Ti and observed the role of twinning in the irreversibility of a martensitic transformation.

**Figure 10:**
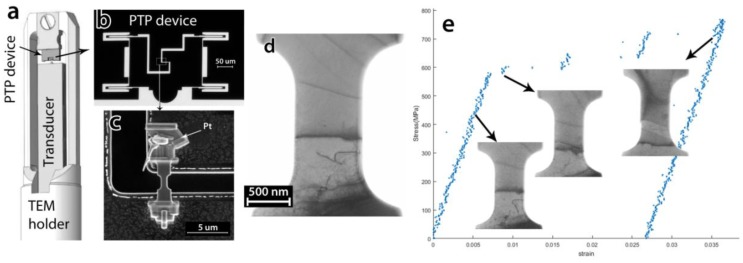
(**a**) Schematic of the PicoIndenter holder; (**b**) TEM BF image of a PTP device; (**c**) SEM image of a tensile sample mounted on a PTP device; (**d**) TEM BF image of a FIB near-defect-free single crystal Ni sample containing only a few dislocations, and (**e**) engineering stress-strain curve of the single crystal Ni sample, the images in the insets are snapshots from the recorded deformation movie, corresponding to the points at the arrows’ origin. Note the strain bursts induced by the operation of the individual SAS (single arm source).

Recent MEMS-based developments have introduced various quantitative nanomechanical testing instruments. The PI 95 Picoindenter holder (Brucker Inc., Billerica, MA, USA), Figure 10a, is a MEMS-based in situ TEM holder which not only provides quantitative mechanical data, but also enables performing different modes of mechanical testing, e.g., tension, compression, bending, and indentation. Such experiments require well-defined micron level sized samples which are often prepared by focused ion beam (FIB) milling. For in situ tensile experiments a so-called push-to-pull (PTP) device, Figure 10b,c is used. The device is designed to convert the compressive loading of the indenter on the semi-circular part, Figure 10b, to a uniaxial tensile loading on the sample mounted in the middle gap, Figure 10c. Tensile testing experiments are of high interest since the uniaxial loading condition is satisfied uniformly during the experiment and interpretation of the mechanical results is straightforward. For instance, Idrissi et al. [105], could investigate the low-temperature rheology of olivine by quantitative in situ TEM tensile tests performed on FIB-prepared micron sized olivine samples, a common mineral in the lithospheric mantle. Heidari et al. [106] used this technique to investigate the mechanical properties of melamine–formaldehyde microcapsules. Idrissi et al. [107] also combined ACOM-TEM and the PTP device to investigate in situ the role of grain rotation in deformed ultrafine-grained Al thin films.

In spite of FIB versatility in small-scale sample preparation, FIB can induce surface defects in metallic or other samples which can not only change the mechanical response of the material [108] but also block the sight to observe the active mechanisms. Samaeeaghmiyoni et al. [109] have developed a new sample preparation method, allowing preparing near defect-free sub-micron metallic single crystals for quantitative in situ TEM tensile testing, Figure 10d. The method also enables designing the sample in terms of crystallographic orientations and imaging conditions as well as pre-selected defects, such as structural boundaries, so that interactions between various defects can be studied. In situ tensile tests on samples prepared by this method revealed new phenomena and demonstrated a direct correlation between the intrinsic mechanical properties and their governing mechanisms. For instance, in situ TEM tensile testing on a single-crystal Ni sample with a very low dislocation density revealed for the first time a hardening mechanism based on the operation of one single arm source (SAS), Figure 10e. The results also shed light on the origin of the intermittent plastic flow characteristics of small-sized single crystals.

### 8.2. LowLoss EELS for Electrical and Optical Properties

EELS also allows to measure and evaluate a number of material properties beyond just the chemistry of a material. Analysing the so-called low-loss range of energies, allows to measure a range of optical and electrical properties of a material, such as the band gap [110], localized plasmon resonances [111,112], phononic excitations, and many others [113,114].

The wealth of information that can be acquired is expanding, and boundaries are constantly pushed.

**Figure 11:**
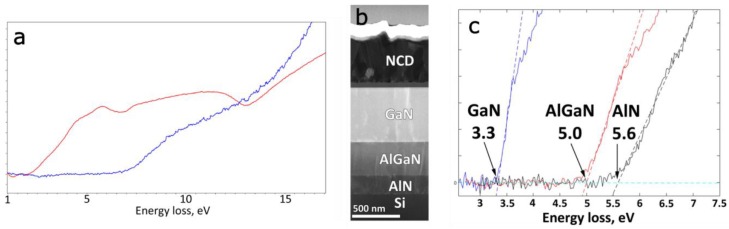
Improvement of band gap measurement. (**a**) Low loss spectrum acquired with the improved setup (blue) contrasted with one acquired in a conventional experiment (red). The distortion of the data caused by the parasitic losses is clearly visible; (**b**) ADF-STEM image of a multilayer sample composed of several wide-band gap semiconductors, and (**c**) recorded spectra clearly showing the onset of the inter-band transitions and, hence, the band gaps, for the different portions of the sample.

The measurement of band gap with EELS is not as commonplace as one might expect. Despite the attractiveness of measuring such properties at the nanoscale, experimental spectra are affected by the presence of “parasitic” losses, mainly the emission of Cherenkov radiation and the excitation of surface guided modes, which blur the onset of the interband transition signal [115]. However, recent advances have presented a practical solution to these problems [116,117,118]. As first suggested by Stöger-Pollach et al. [118] and then developed in detail by Korneychuk et al. [117], the large difference in scattering angles between the interband and “parasitic” signals can be exploited by collecting electrons scattered away from the forward direction within a carefully chosen angular range that allows the faithful recovery of band gap while avoiding the disturbances (Figure 11). The further addition of a custom designed annular aperture in the illumination system of the TEM allows to realize this scattering geometry while avoiding any asymmetry that could be introduced by going off-axis [117,118].

Another field where recent advances have greatly broadened the information that can be obtained from EELS is the mapping of localized surface plasmon resonances (SPRs). While TEM already has an important role in the analysis of plasmonic resonances, a great deal of information is hidden by the conventional approach. Only the squared field intensity is mapped, and then only in the component parallel to the optical axis [119,120]. This constitutes a strong limit to perform, e.g., reliable and direct plasmon field tomography, or effectively retrieve the surface charge distribution, the real field direction, or the dynamical behaviour. A wealth of recent developments however have now broadened the scope significantly. Pulsed electron sources have allowed to observe the dynamical behaviour of reproducible phenomena with femtosecond resolution [121]. Even more recently, by analysing the angular distribution of inelastically scattered electrons, effectively joining EELS and momentum-resolved STEM, Krehl et al. have measured the thus-far elusive transverse field produced by localized surface plasmon resonances [122].

Finally, it has also been shown that the dynamic electrical potential of the SPRs has an effect on the electron beam. Just like an electrostatic potential causes a phase shift on an electron beam, the dynamical one causes a phase shift in the inelastically scattered part of the beam. This has been exploited by Guzzinati et al., manipulating the complex wave function of the impinging electron beam, to directly test the charge symmetry of plasmonic modes [123]. While such experiment are cumbersome with current methods, development of more flexible techniques to manipulate the electron beam’s phase is progressing rapidly and a first prototype of a programmable phase plate has been presented by Verbeeck et al. [124]. Such developments are expected to help make phase-based experiments more common [123,124].

## Supplementary Materials

The following is available online at http://www.mdpi.com/1996-1944/11/8/1304/s1. Video S1 showing the time series of the [010] HAADF-STEM images for Bi_10_Ti_7_Fe_6_O_38_ and unveiling the cooperative displacements of Bi atomic columns (A cations) in the structure. Adjacent A2 columns displace in opposite directions that is visible as a rotation of the A2 atomic column pairs about the b axis. The rotation of the A2 pairs induces displacements in the neighboring A1 columns. The displacements in the A3 atomic columns are very subtle and difficult to distinguish because of scan distortion of the HAADF-STEM images. Reprinted with permission from [42], copyright 2016 American Chemical Society.
